# Antibody responses to avian influenza viruses in wild birds broaden with age

**DOI:** 10.1098/rspb.2016.2159

**Published:** 2016-12-28

**Authors:** Sarah C. Hill, Ruth J. Manvell, Bodo Schulenburg, Wendy Shell, Paul S. Wikramaratna, Christopher Perrins, Ben C. Sheldon, Ian H. Brown, Oliver G. Pybus

**Affiliations:** 1 Department of Zoology, University of Oxford, Oxford OX1 3PS, UK; 2 Edward Grey Institute, Department of Zoology, University of Oxford, Oxford OX1 3PS, UK; 3 Department of Virology, Animal and Plant Health Agency (APHA), Weybridge KT15 3NB, UK

**Keywords:** avian influenza, virus, immunity, bird, age, lifespan

## Abstract

For viruses such as avian influenza, immunity within a host population can drive the emergence of new strains by selecting for viruses with novel antigens that avoid immune recognition. The accumulation of acquired immunity with age is hypothesized to affect how influenza viruses emerge and spread in species of different lifespans. Despite its importance for understanding the behaviour of avian influenza viruses, little is known about age-related accumulation of immunity in the virus's primary reservoir, wild birds. To address this, we studied the age structure of immune responses to avian influenza virus in a wild swan population (*Cygnus olor*), before and after the population experienced an outbreak of highly pathogenic H5N1 avian influenza in 2008. We performed haemagglutination inhibition assays on sampled sera for five avian influenza strains and show that breadth of response accumulates with age. The observed age-related distribution of antibody responses to avian influenza strains may explain the age-dependent mortality observed during the highly pathogenic H5N1 outbreak. Age structures and species lifespan are probably important determinants of viral epidemiology and virulence in birds.

## 1. Introduction

The ability to rapidly respond to viral infection via recognition of previously encountered antigens is a critical part of the host adaptive immune response. Viruses such as influenza A virus, HIV-1 and norovirus effectively evade host immune systems because of their capacity to evolve novel antigenic variants. Existing immunity within a host population drives the emergence and spread of new antigenic strains by selecting for viruses with novel antigenic sites that avoid host immune recognition. Understanding the landscape of immunity within a population is consequently fundamental to understanding the epidemiological dynamics of antigenically diverse pathogens.

Influenza A viral dynamics in human and avian hosts are fundamentally different. In humans, existing viruses are typically replaced by antigenically different strains belonging to the same virus subtype. Replacement by a strain of a different subtype (‘antigenic shift’) occurs only every 10–50 years [1]. The return of an antigenically similar strain also occurs comparatively rarely: antigenic similarities noted between the H1N1 strains circulating in 1918 and 2009 were remarkable [2,3], because strains separated by more than 2–8 years are usually considered to be antigenically distinct [4–6]. In bird populations, many genetically diverse subtypes can coexist [7]. Modelling studies have investigated whether differences in influenza virus dynamics between species of different lifespans are in part a consequence of the faster rate at which immunologically naive hosts are replenished in shorter-lived species compared with long-lived species [8,9]. Such studies assume that immunological memory to a specific antigen can last for the lifetime of the host and thus the breadth of response to different antigenic strains increases as an individual ages. While this seems probable for influenza A viruses (AIV) in humans [10–13], little is known about the acquisition and retention of immunity to influenza A viruses in wild birds, which form the primary reservoir of avian AIV [14,15]. Understanding the accumulation of adaptive immunity in wild birds is important for understanding the ecology of AIV viral prevalence and transmission dynamics, including predicting in which species novel strains are most likely to emerge.

Much of our knowledge about the acquisition of adaptive immunity to AIV in birds has been derived from experimental inoculation of immunologically naive, domesticated waterfowl [16–19]. It has been particularly challenging to quantify changes in acquired immunity with age, because the exact ages of wild-caught birds are usually unknown or because lifespans of many domestic bird species that harbour AIV are too short for meaningful patterns to be discerned. An experimental study of wild-caught gulls reared in captivity showed that exposure to AIV results in increased protection against that strain for at least 1 year [19], suggesting that continual exposure to AIVs could result in better protection with increased age. However, while experimental studies provide important insights under controlled conditions, they may not adequately describe long-term acquisition of immunity in wild populations that are exposed to a diverse range of AIV subtypes over prolonged periods and to other sources of mortality.

Previous studies have indicated that acquired adaptive immunity may shape the observed incidence of AIV in wild birds of different ages. For example, juvenile birds from many anseriform species (including ducks, geese and swans) are less likely to carry antibodies against AIV than adult birds [20–27]. Juveniles also have higher viral prevalence than adult birds [14,20,28,29], suggesting that immunologically naive juveniles are more susceptible to infection or shed virus for longer than older birds. At the population level, seasonal peaks in viral prevalence have been observed following hatching, and attributed to the immunological naivety of the unfledged birds [29]. Immunologically naive birds that are challenged by an AIV have similar shedding patterns and probability of seroconversion, regardless of bird age [19]. It is therefore likely that the age-related patterns of seroprevalence and viral prevalence observed in the wild result from birds gaining immunity to AIV with continual exposure throughout the lifespan, rather than changes in immune function specifically resulting from ageing.

Studies of adaptive immunity in wild bird populations may generate data with limited resolution, for two reasons. First, with few exceptions [25,26], information on the exact age of the wild birds being studied is unavailable, and thus age must be reduced to a binary variable (adult versus juvenile). Second, immune responses to AIV in wild birds are often characterized using the presence or absence of antibodies to the AIV nucleoprotein (NP), rather than to a specific haemagglutinin (HA) or neuraminidase (NA) type. The high sequence conservation of NP among AIV strains means that a single NP-antibody test can easily identify whether a bird has been infected with AIV, but cannot distinguish among acquired immune responses to specific subtypes.

It is not known whether studies that use NP-antibody tests are failing to detect subtype-specific variation in immunity among birds of different ages. Haemagglutinin (HA) is the most abundant of the two AIV surface proteins, and antibodies raised against HA are central to the adaptive immune defence [30]. These antibodies neutralize the virus by binding to the HA protein, preventing virus attachment and cell entry. Experimental and natural infections of waterfowl have shown that infection with a given HA type can induce protective immune responses to that same HA type (homosubtypic immunity) and may also cause weaker immunity to different HA types [16–18,31–34] (heterosubtypic immunity). Haemagglutination inhibition (HI) titre is often correlated with the strength of protection against AIV when vaccine and challenge antigens are similar [35–38]. If birds form a long-term immunological memory of encountered HA antigens, then birds exposed to many strains may be protected against a wider range of viruses than birds that have only ever been exposed to a few strains (e.g. younger birds). Whether the breadth of responses to different AIV HA types increases with age has not been investigated [8,9].

The Abbotsbury Swannery in Dorset, UK, harbours a semi-habituated population of wild mute swans (*Cygnus olor*), a long-lived species that has been subject to long-term study [39,40]. All swans born into the colony are marked after hatching, so birth dates are known for most individuals. Birds are vent-sexed at hatching and by breeding behaviour. Birds that immigrate into the population are aged by plumage where possible and sexed during regular ringing events. The population is thus suitable for testing hypotheses about age-related acquisition of immunity. In winter 2007/2008, an outbreak of highly pathogenic avian influenza (HPAI) H5 occurred in the population [41]. During the outbreak, there appeared to be a predominance of young birds among the H5N1-positive dead birds [26]. Although this trend was not statistically robust owing to small sample sizes, evidence from experimental challenge studies indicated that birds with prior exposure to AIV survive infection with pathogenic AIV and shed virus for shorter periods than immunologically naive birds [31,32,42]. Because younger, immunologically naive birds were more affected in the 2008 H5N1 outbreak, we hypothesize that antibody responses to related LPAI viruses were lower in younger than older birds prior to that event.

Here, we report the first investigation of the age structure of immunity to different AIV subtypes in a wild bird population for which bird ages are known. Blood samples from our study population were obtained in 2007 and 2008, either side of the H5N1 HPAI outbreak. To explore the pattern of immunity to AIV, we performed HI assays for five HA antigens (belonging to three HA types), and undertook statistical analysis to determine whether antibody responses to specific HA types and/or the breadth of antibody responses are associated with age in the population. We consider whether a lack of pre-existing immunity to different LPAI subtypes could have resulted in the raised mortality among young birds during the H5N1 outbreak in the winter of 2007/2008.

## 2. Methods

### (a) Study population

The Abbotsbury Swannery in Dorset, UK (50.6537°N, 2.6028°W), harbours a semi-habituated population of wild mute swans (*C. olor*) that has been subject to long-term study [39,40]. Following hatching, a small proportion of breeding pairs and cygnets are placed for four months in pens. All other swans have freedom of movement and mix naturally with other species. Survival rates of first-year and adult birds are similar to those across the UK [43,44], but the survival rates of birds in their second and third years are slightly higher at Abbotsbury [39]. Overall longevity is similar to elsewhere in the UK [44]. The population is unusually large and varies seasonally (in July 2007, population size was approximately 900–1000). Approximately 150 breeding pairs nested at Abbotsbury in 2007 and 2008. Supplementary food is provided from spring to autumn. A fence around the site reduces terrestrial predation.

### (b) Population sampling

Blood samples were collected from swans at Abbotsbury during July 2007 and August 2008 (UK Home Office licences PPL 80/1944 and PPL 30/2572, respectively), as described previously [26]. Where a bird was sampled in both years (11 birds total), a single sample was excluded randomly, so that birds were not repeated across years in the dataset, leaving 63 samples from 2007 and 95 samples from 2008. Birds with repeated sampling were considered in a separate analysis. Age and/or sex was unknown for a small proportion of birds (3.2% of birds had unknown age only, 8.9% had unknown sex only and 1.9% had both unknown age and unknown sex), and these birds were removed from analyses where appropriate. Sampling was intentionally slightly biased towards older birds, and the age structure sampled across both years was similar (electronic supplementary material, figure S1*a*). Seropositivity prevalences reported here should therefore not be interpreted as if the sample was random.

### (c) Serological assays

HA antigens from five different viruses were chosen for HI assays. Antigen choice was motivated by an interest in protection against mortality from HPAI H5N1 infection following previous exposure with LPAI strains. Because protective immunity acts more effectively within than between HA groups, we chose viruses from HA clade 1 (the clade that includes H5 viruses). Within this clade, we chose HA types that had been experimentally associated with reducing the severity of subsequent HPAI H5N1 infections (H5 and H9 [31,34,45,46]) or were antigenically close to H5 viruses (H6). H6 viruses were among the most common viruses in European waterfowl around the sampling period of 2007/2008, particularly in geese and swans [28,47,48]. LPAI H5 viruses were very common, comprising 4.9–8.4% of LPAI infections in European wild birds [47,49]. H9 viruses were rarer than H5 or H6, but were not uncommon during this period in several species and locations [27,47,48] and were interesting because of their association with protection against H5N1 HPAI infection.

The Abbotsbury swans had not been screened for AIV prior to 2007/2008, so it was unknown which AIVs, other than HPAI H5N1, had circulated in the population. We therefore selected antigens that were broadly cross-reactive with several modern strains of the same HA type (identified during years of serological testing at the UK Animal and Plant Health Agency). The chosen antigens were H5N1 A/mute swan/England/26-20/2008 (the HPAI outbreak strain at Abbotsbury), H5N2 A/ostrich/Denmark/72420/1996, H6N8 A/ostrich/South Africa/946/1998, H9N2 A/turkey/USA Wisconsin/1966 and H9N9 A/knot/England/SV497/2002. Many of these antigens are commonly used in European Influenza Reference Laboratories, and the assays correspondingly well established.

HI assays were conducted to determine the presence of antibodies for specific HA types according to standard methods [50]. Sera were pre-treated with receptor-destroying enzyme (RDE; one volume of serum to five volumes of RDE) before heat-inactivation at 56°C for 30 min. Inactivated sera were treated with chicken red blood cells (RBCs). Serial twofold dilutions of the sera were prepared from a starting dilution of 1 : 10. HI assays were performed using a 1% suspension of chicken RBCs in PBS. Owing to low volume of some samples, all five assays were performed only on 61 and 88 of the 2007 and 2008 samples, respectively. Four HA units of antigen per 25 µl were added to the serial dilutions. Haemagglutination titres of greater than or equal to 20 were considered seropositive, based on the OIE recommended cut-off of 1/16 [50].

### (d) Statistical analyses

Cumulative link models were used to assess the effects of age on breadth of the antibody response. ‘Breadth’ was calculated as the total number of subtypes a bird was seropositive for. To ensure that the specific choice of HA antigen included had no significant effect on the estimated relationship between breadth and age, we calculated breadth of responses based on the presence or absence of a serological response to (i) H5N2, H6N8, H9N2 (henceforth referred to as dataset A), (ii) either strain belonging to the same HA type (dataset B), (iii) both strains belonging to the same HA type (dataset C), (iv) H5N1, H6N8 and H9N9 (dataset D), and (v) H5N2, H6N8 and a response to both H9N2 and H9N9 (dataset E). Age, sex and sample year were included as fixed factors in the statistical model. Likelihood ratio tests of cumulative link models were used to compare models with and without interaction terms between all pairs of factors. Interaction terms were included if a model with the interaction term had a probability of less than 0.05 of being a better explanatory model than one without the interaction, based on likelihood ratio tests. Two-sided tests were used to generate all *p*-values.

Generalized linear models with a gamma-distributed response were used to assess the correlation between breadth of response in a sample and the result of the AIV NP-ELISA test on that sample (data for the latter obtained from [26]). The effect of age, sex and sample year on the NP-ELISA result was also tested.

Generalized linear models with a binomial response and logit link were used to determine whether the probability of being seropositive for each individual subtype increased with age. Age, sex and sample year were included as fixed factors, and interaction terms were tested using chi-squared tests of the difference in deviance between the two models. An interaction term was included if the model with the interaction term had a probability of less than 0.05 of being a better explanatory model than the model without the interaction.

Samples that tested seropositive (greater than or equal to 20) for each antigen were separated into two groups: those with titres less than 40 and those with titres of 40 or more. Generalized linear models with a binomial response and logit link were used to determine whether the magnitude of positive titres varied with age. Sex and sample year were included as additive predictors. Binning titres into two categories was considered more appropriate than using raw titres in ordinal regression models, as there were few titres of greater than or equal to 80 for all tested antigens except H9N9.

To analyse whether immune responses were stable across consecutive years, analyses were conducted on eleven birds sampled in both 2007 and 2008. Wilcoxon signed-rank tests were conducted on titres from each assay, and separately on the overall breadth of response, to determine whether there was an increase or decrease by year. McNemar tests were used to determine whether seropositivity in the population for each HI assay increased or decreased over the year. Ordinal logistic regression was used to test whether an increasing breadth of response between years was associated with a decrease in NP-ELISA result.

## 3. Results

### (a) Seroprevalence varies by subtype

**Figure 1.**
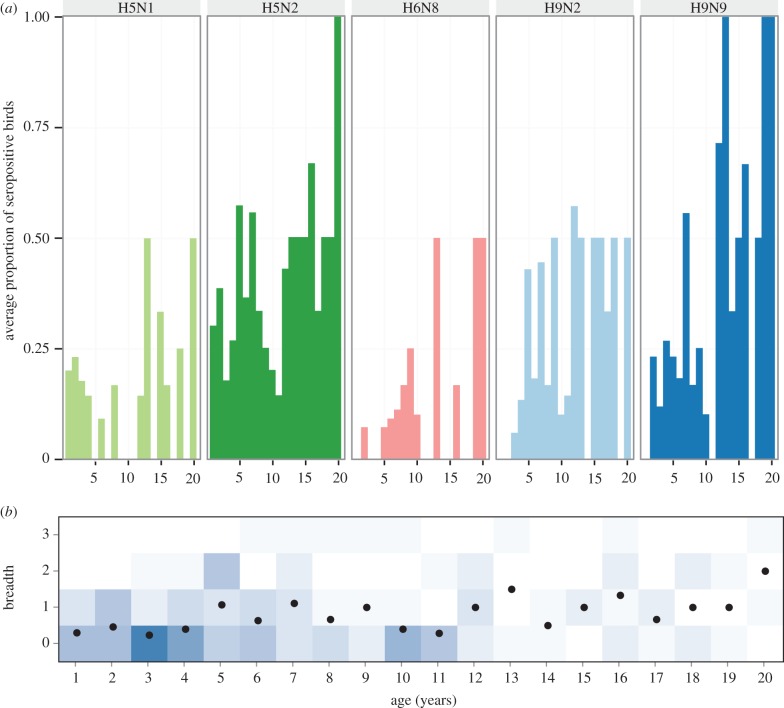
Immune responses of birds in the population. (*a*) Proportion of birds of different ages that respond to each strain. (*b*) Breadth of immune response (measured as the number of serotypes responded to from dataset A; see Methods) for birds of different ages. The darker blue the square, the more birds of each age respond to each number of subtypes. The mean number of subtypes responded to at each age group is plotted as a black dot. (Online version in colour.)

HI assays were conducted for five AIV antigens belonging to three HA types. Seroprevalence in the (non-random) sample of birds varied according to subtype, with H5N2 being most seroprevalent in both sample years, H5N1 least seroprevalent in 2007 and H6N8 least seroprevalent in 2008 (table 1 and figure 1*a*). Raw data files are available in electronic supplementary material, file S1.

**Table 1.** Population seroprevalence (number birds positive) for each test antigen.

sampling date (birds sampled)	H5N1	H5N2	H6N8	H9N2	H9N9
July 2007 (61)	6.56 (*n* = 4)	39.3 (*n* = 24)	8.20 (*n* = 5)	21.3 (*n* = 13)	21.3 (*n* = 13)
Aug 2008 (88)	15.9 (*n* = 14)	35.2 (*n* = 31)	6.82 (*n* = 6)	23.9 (*n* = 21)	29.5 (*n* = 26)

### (b) Subtype breadth increases with age

**Figure 2.**
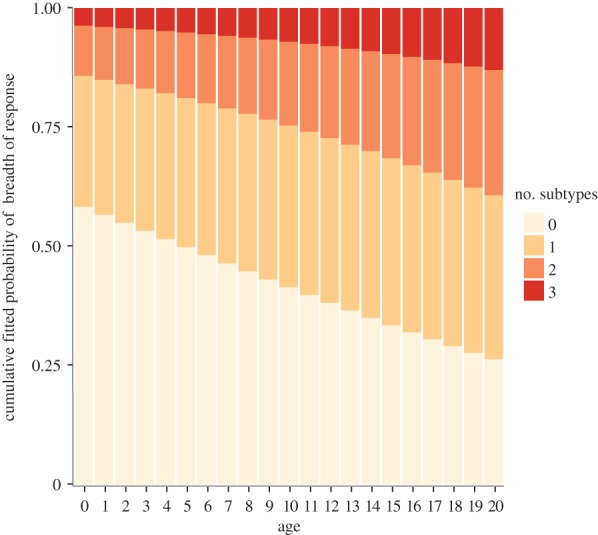
Cumulative fitted probabilities of effect of age on number of strains to which a swan exhibits a response (breadth of response) as determined by cumulative link models. Sex and sample year were included in the model, but do not significantly affect the probability of having a response, so for simplicity, data are shown for female birds in 2007. Breadth of response details the number of subtypes birds respond to (dataset A, including H9N2, H5N2 and H6N8). (Online version in colour.)

As birds age, they are increasingly more likely to respond to a wider variety of HA types (figure 1*b*). Owing to the possibility of correlated responses to multiple H9 or H5 types, we explored a variety of dataset combinations (see Methods). Likelihood ratio tests of cumulative link models suggested that inclusion of interaction terms did not significantly improve the model, so only the main effects of sex, age and sample year were included. In all of the tested datasets A–E except dataset C (see Methods), older birds were significantly more likely to have a broader response to different AIV HA types than younger birds (electronic supplementary material, table S1 for *p*-values and coefficients of the cumulative link model). When breadth is calculated as response to H9N2, H5N2 and H6N8 subtypes (dataset A), the probability that a bird exhibits responses to X or more HA types increases by a factor of 1.1 with every extra year of age (*p* < 0.05; figure 2). Male birds were more likely to have narrower responses to AIV than female birds in all datasets, but this was only significant for a model where breadth was calculated from either dataset A or E.

The breadth of response observed in a sample was correlated with the raw result of the NP-ELISA for that sample, such that a larger breadth of response was associated with lower ELISA values. This result was robust to the way in which breadth was calculated (electronic supplementary material, table S4). Age also correlated with NP-ELISA score, which is unsurprising as age is collinear with breadth of response in our data (electronic supplementary material, table S5).

### (c) Effect of age, sex and sample year on each individual subtype

Generalized linear models were used to determine whether age was associated with responses to each individual subtype. Age, sex and sample year were included as fixed factors. For H5N1, H6N8 and H9N9 and H9N2, no interaction terms significantly increased the predictive power of the model. An interaction effect between sample year and age was included for H5N2.

**Figure 3.**
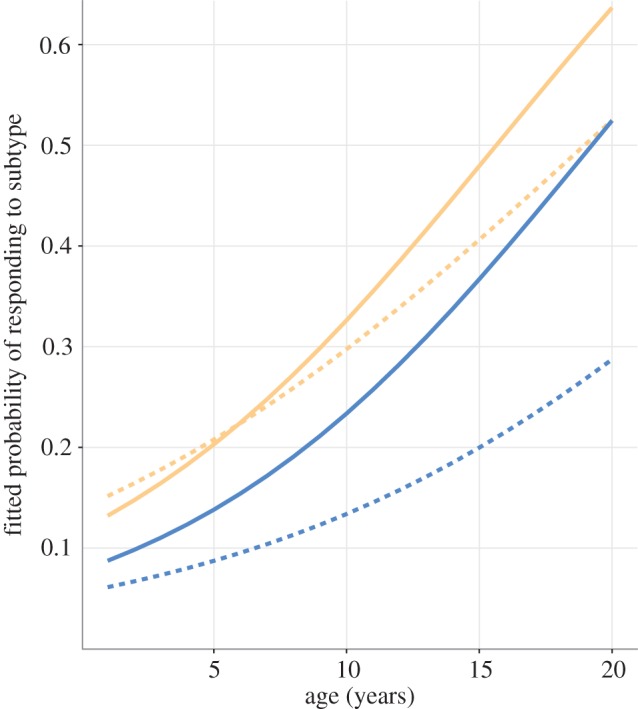
Fitted probability of effect of age and sex (females; pale orange, males; dark blue) on response to H9N9 (solid line) and H9N2 (dashed line) as determined by GLM with logit link. Sample year was included in the model, but does not significantly affect the probability of having a response, so for simplicity, data are shown for 2007. (Online version in colour.)

When individual subtypes were considered, age was found to be a significant predictor of response to H9N2, H9N9 and H5N2 subtypes (*p* < 0.05, *p* < 0.0005 and *p* < 0.05, respectively; figure 3; electronic supplementary material, table S2). For every extra year of age, the odds of responding to H9N2 increased by 1.10 times, for H9N9, 1.14 times and for H5N2, 1.17 times. Males were significantly less likely to be seropositive for H9N2 (odds 0.37, *p* < 0.05). While males were also slightly less likely to be seropositive for H9N9, H6N8 and H5N2, these effects were not significant.

**Figure 4.**
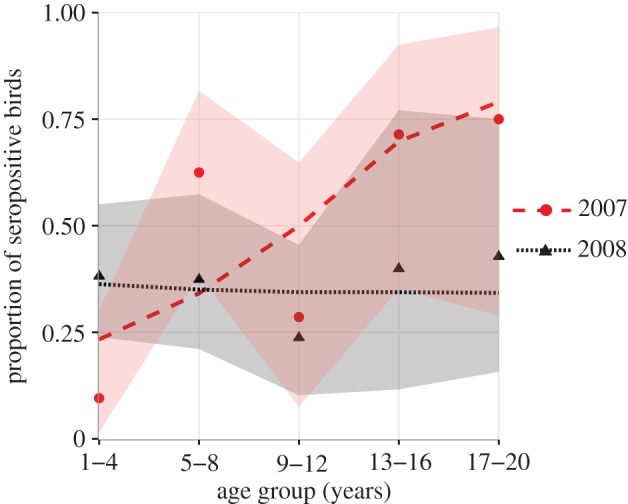
Proportion of birds observed to respond to H5N2 in five age groups for each sample year (dots), with 95% CIs for proportions shaded (2007, pale red; 2008, dark grey). Lines represent the mean proportion of birds in each age category expected to respond to H5N2, based on the fitted probability of effect of age and sample year (2007, red, wide-dash; 2008, black, narrow-dash) on response to H5N2 as determined by GLM with logit link. As the inclusion of sex in the model does not significantly affect the probability of having a response, for simplicity, expected number of birds is based on the fitted probability estimated for a model without a sex term. (Online version in colour.)

Notably, antibody responses to H5N1 were significantly higher in 2008 than in 2007. The sampling year significantly affected the relationship between age and response to subtype H5N2. In 2007, older birds were more likely to respond to H5N2 than younger birds, whereas in 2008, there was no significant relationship with age (figure 4). When an interaction term between sampling year and age was fitted to the H5N1 data, we still did not observe a significant interaction effect. Age and sex had no significant relationship on the probability of response to H5N1. Sample year did not affect the probability of response to H9N2 or H9N9, and age, sample year and sex had no significant effect on the probability of response to H6N8. For seropositive samples from all tested antigens, there was no effect of age, sex or sample year on the magnitude of the titre.

### (d) Longitudinal sampling in birds from both years

Data were available from both 2007 and 2008 for 11 birds. Responses to each HA antigen were not stable between years (electronic supplementary material, figure S2), but McNemar tests did not detect a significant increase or decrease in the number of seropositive birds for each assay by year (*p* > 0.05 for all tests). Wilcoxon signed-rank tests indicated that raw titres for every HA type, and also for breadth of response, showed no significant increase or decrease by year (*p* > 0.05 for all tests). The failure to detect a population-level effect of increasing breadth of response with increasing age between 2007 and 2008 was probably due to (i) the small sample size of 11 birds and (ii) the small estimated effect of age on breadth of response, which only becomes apparent across multiple years. A decrease in NP ELISA values between years was associated with an increase in response breadth, but this was not significant (datasets A–E; electronic supplementary material, table S3).

## 4. Discussion

Studies of AIV seroprevalence have shown that ‘adult’ birds (typically individuals 1 or 2 years after hatch-year, depending on species) are more likely than ‘juvenile’ birds (sampled during or 1 year after hatch-year) to harbour antibodies targeted at AIV NP [20–27]. Here we show that not all birds defined as seropositive for AIV by NP antibody presence have equivalent levels of protection against AIV. Instead, older birds are increasingly more likely to produce antibodies directed at a wider range of HA types. Our data suggest that mute swans have a long-term immune memory, possibly similar to that exhibited by humans, in which memory B cells produced during a primary infection are reactivated following further infections of a related HA type [10–13]. The ability of wild waterfowl to form lifelong immune memory in response to naturally encountered infections supports key assumptions made in modelling studies of AIV infection dynamics [8,9].

The pattern observed here could be generated by at least two non-exclusive mechanisms. In the first scenario, a high proportion of birds is exposed to AIV each year, but younger birds are less likely to form long-term immune memory to the strains than older birds. However, while increases in the ability to form immune memory with age have not been investigated, such gains in immune function seem improbable given that birds appear to undergo immune-senescence as they age [51–54]. In the second scenario, a low proportion of birds is exposed to different AIV HA types every year, but all age groups are equally likely to form a long-term immune response. The prevalence of AIV in healthy wild mute swan populations is low (typically less than 3% [15,27,47,48,55]), with most infections observed in juveniles (approx. 6% prevalence) [27]. Experimental evidence in mallards (*Anas platyrhynchos*) suggests that birds are infected with approximately half of all HA types (6/11) circulating in the population within the first 2 years of life [33]. On average, the AIV prevalence in swans is thought to be considerably lower than the prevalence found in wild mallards [15,49], so it is plausible that the number of infections with different HA types per swan per year is also lower than in mallards. If so, swans would encounter a relatively low number of HA types each year and would form a long-term HA immune response to at least a proportion of these viruses. This scenario is compatible with the observations in our study population of high seropositivity for AIV NP [26] and the accumulation of HA-type-specific responses throughout life.

Several previous studies of AIV seroprevalence have found that male birds are less likely than females to be serologically positive for AIV [20,24,27,29]. However, other studies have shown the opposite trend [25]. Our data suggest that male birds consistently had lower levels of NP antibodies and a slightly narrower response than female birds, but the significance of this result varied depending on which strains were used to calculate breadth. It has been suggested that sex-specific seroprevalence might result from different breeding behaviours of each sex [25] or inherent differences in immune response or antibody persistence [24,51,52]. We find no evidence to support the hypothesis that age affects the relationship between sex and seroprevalence, which might be expected if there were an effect of breeding status.

Among those birds that did exhibit an antibody response, we found no evidence for an effect of age on the magnitude of HA antibody titre, suggesting that the responses of younger birds to our test antigens are as strong as those of older birds. Hence, any age-related differences in immunity derive only from the presence or absence of specific responses. It is unclear whether increasing seropositivity with age corresponds to increasing protection against the effects of AIV infection. As well as humoral immunity, cellular immunity may contribute to faster recovery and reduced viral shedding following infection, via the recognition of AIV-infected cells by cytotoxic T-lymphocytes (CTLs). Although little work has characterized CTL responses in birds, it has been shown *ex vivo* that chicken CTLs can target AIV NP and HA [56,57]. Recognition of the conserved NP could result in cross-protection among AIV viruses, including those with antigenically distinct HA types [58,59]. In our study, we defined seropositive birds as those with titres greater than or equal to 20. This is substantially lower than the threshold recommended by the OIE for vaccine-induced protection against mortality (greater than or equal to 32) or reduced viral shedding (greater than or equal to 128) [50]. However, several studies suggest that protection may be afforded by lower titres than those specified by OIE, including a threshold of 20 or less for a good probability of protection against mortality from HPAI infection, and of 40 or more for prevention of viral shedding in most infected birds [36–38]. Secondary infections with LPAI viruses show significantly reduced shedding even when the original antibody HI response is weak (less than 20) [16]. If protection at low titres is caused by humoral immunity, we would expect that increasing seropositivity with age would result in increasing protection against the effects of AIV infection. Conversely, if protection at low HI titres results from immune cross-protection against conserved proteins, there may be little protective benefit of an increasing number of weak responses against a larger number of HA types.

Interestingly, our data suggest that older birds testing seropositive for previous infection by NP-ELISA typically have stronger responses in that NP-ELISA assay than seropositive younger birds. Anti-NP antibodies are non-neutralizing, but have been shown to contribute to protective cross-subtype immunity in mice [60,61]. If NP antibodies are found to contribute to protective immunity in birds, then the age-correlated differences in NP antibody levels observed here may result in differences in immune protection against AIV with age. Research into the relative importance of humoral and cellular responses against AIV when HA antibody responses are low is needed to understand how age, immune protection and serological breadth are related.

The natural outbreak of HPAI H5N1 in the Abbotsbury population in winter of 2007/2008 enables us to consider whether age-specific immunity may have had an effect on, or been affected by, the dynamics of that outbreak. The only previously identified case affecting wild birds in the UK involved a single whooper swan (*Cygnus cygnus*) in Scotland in 2006 [62]. It is therefore very unlikely birds at Abbotsbury had encountered AIV from the HPAI H5N1 lineage prior to the start of the outbreak in late 2007. Consequently, the low level of reactivity to HPAI H5N1 that was observed in sera sampled in summer 2007 (6% of birds) may indicate a low level of protective immunity in the population as a result of previously encountered LPAI viruses. Interestingly, we observed a significant population-level increase in HPAI H5 antibody prevalence after the outbreak. This is consistent with a scenario in which a proportion of birds developed antibody responses against the HPAI H5N1 virus following undetected infections during the outbreak. Experimental work suggests that waterfowl previously exposed to an LPAI virus are more protected against the effects of subsequent exposure with H5N1 [18,31,42,46]. Our data suggest that older birds were more likely to have such pre-existing antibodies than younger birds. We speculate that this could have caused the higher mortality observed in younger birds (3 years old in January 2008) during the 2008 HPAI H5N1 outbreak [26].

There are several caveats to our study. First, the samples used here were collected exclusively during July and August, and hence we cannot determine if seasonality affects any of the relationships that we report here. Active infection rates appear to fluctuate seasonally in wild waterfowl, and some evidence suggests that serological responses may also vary seasonally [20]. Second, while our results are robust to a choice of H5 and H9 antigens, our choice of strains and HA types may have affected the results. Despite focusing on only five strains, we found a significant increase in breadth of humoral response with age using just three HA types. Consequently, we suspect our results are conservative and that the age-dependent effect would be stronger if more antigens had been tested. However, we cannot rule out that testing using a larger panel of viruses isolated over a long time in the test population might affect the results. Third, the extent to which our results can be generalized to other long-lived, wild waterfowl is unknown. Swans at Abbotsbury may experience higher levels of AIV exposure than other mute swans owing to the unusually large population size, and different environmental and nutritional stresses (see Methods). Despite this, we think that it is unlikely that the capacity to form long-term immune memory would be qualitatively different in other mute swan populations or related species.

In summary, we find that older wild mute swans are more likely to have broader antibody responses against AIV, and hence may be protected against a wider range of viruses. Our data suggest that immune memory in this population, and perhaps also in other waterfowl populations or species, is long-lasting. The profile of existing immunity within a population almost certainly affects the demographic impact of new viral infections such as HPAI H5N1. Further, accumulation of antibody responses throughout life probably generates differences in the epidemiology of AIV among host species with different lifespans. A more complete understanding of influenza dynamics will require long-term screening of active infection and serological immunity in other well-studied animal populations in nature.

## Supplementary Material

Supplementary Figure 1

## Supplementary Material

Supplementary Figure 2

## Supplementary Material

Supplementary Figure 3

## Supplementary Material

Supplementary Tables

## Supplementary Material

Supplementary Data File 1

## Data accessibility

Raw data are available in electronic supplementary material, file S1.
