# Supplementary material for: Antibody responses to avian influenza viruses in wild birds broaden with age
Source: Proc Biol Sci. 2016 Dec 28;283(1845):20162159. doi: 10.1098/rspb.2016.2159 (PMC5204166; doi:10.1098/rspb.2016.2159)
Supplement: Supplementary Figure 1 [file rspb20162159supp1.pdf]

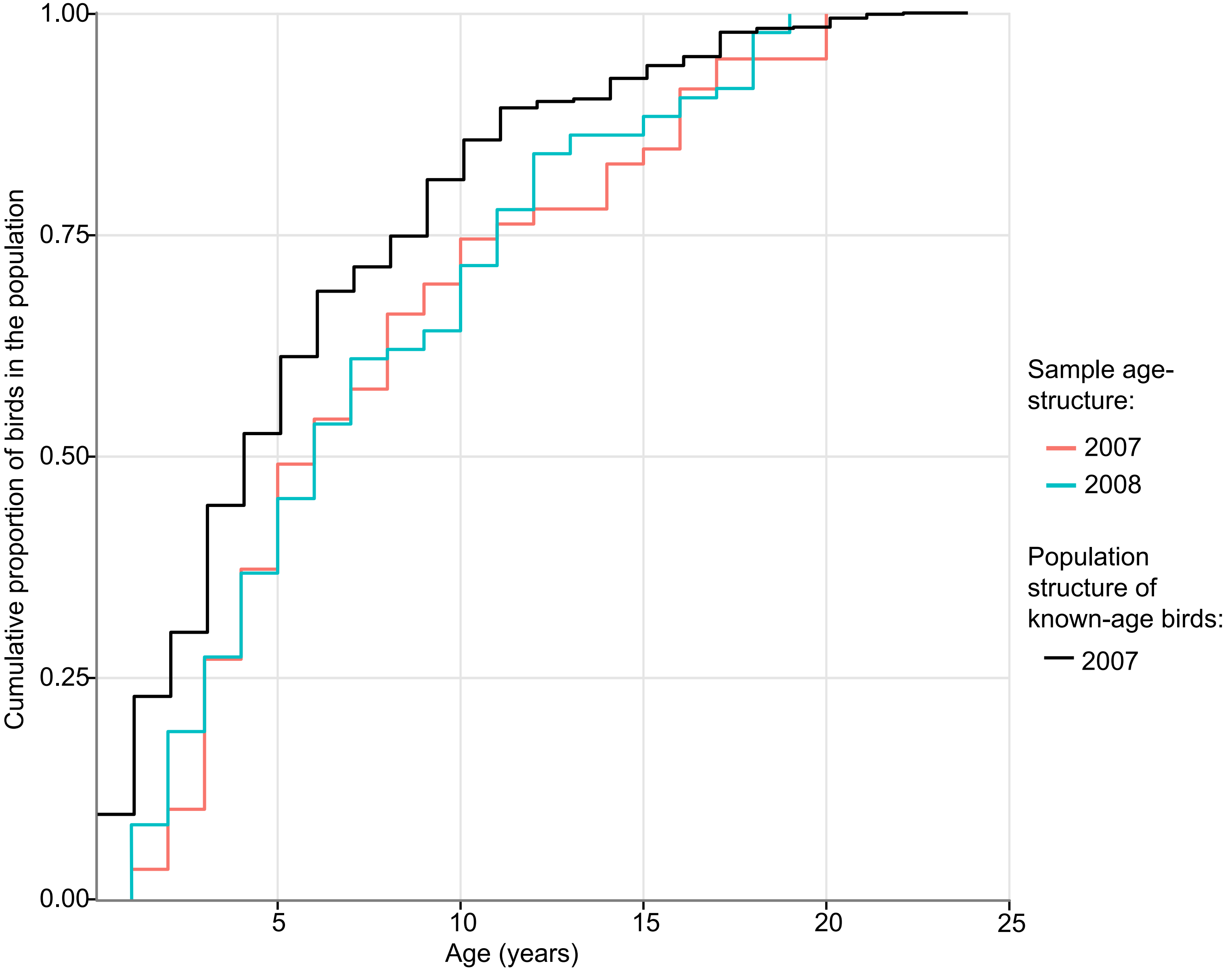


Supplementary Figure 1: Cumulative proportion of birds sampled during each sampling year (red and blue lines) and in the population (black lines). The population age-structure is based on birds with known ages caught or known to be alive (breeding birds) during a biennial catch of all adult birds on July 21st 2007. Because cygnets born during 2007 at Abbotsbury were not caught on this day, the age-structure also includes all Abbotsbury cygnets that were ringed after July 2007 (typically during late August to mid October). Cygnet mortality during late summer and prior to ringing may mean that the number of cygnets alive during July 2007 is slightly underestimated here. In total, approximately 80% of the birds believed to be in the population on July 21st 2007 had known ages. Birds of unknown ages typically entered the population as adults and could not be aged.
