# Supplementary material for: Antibody responses to avian influenza viruses in wild birds broaden with age
Source: Proc Biol Sci. 2016 Dec 28;283(1845):20162159. doi: 10.1098/rspb.2016.2159 (PMC5204166; doi:10.1098/rspb.2016.2159)
Supplement: Supplementary Figure 2 [file rspb20162159supp2.pdf]

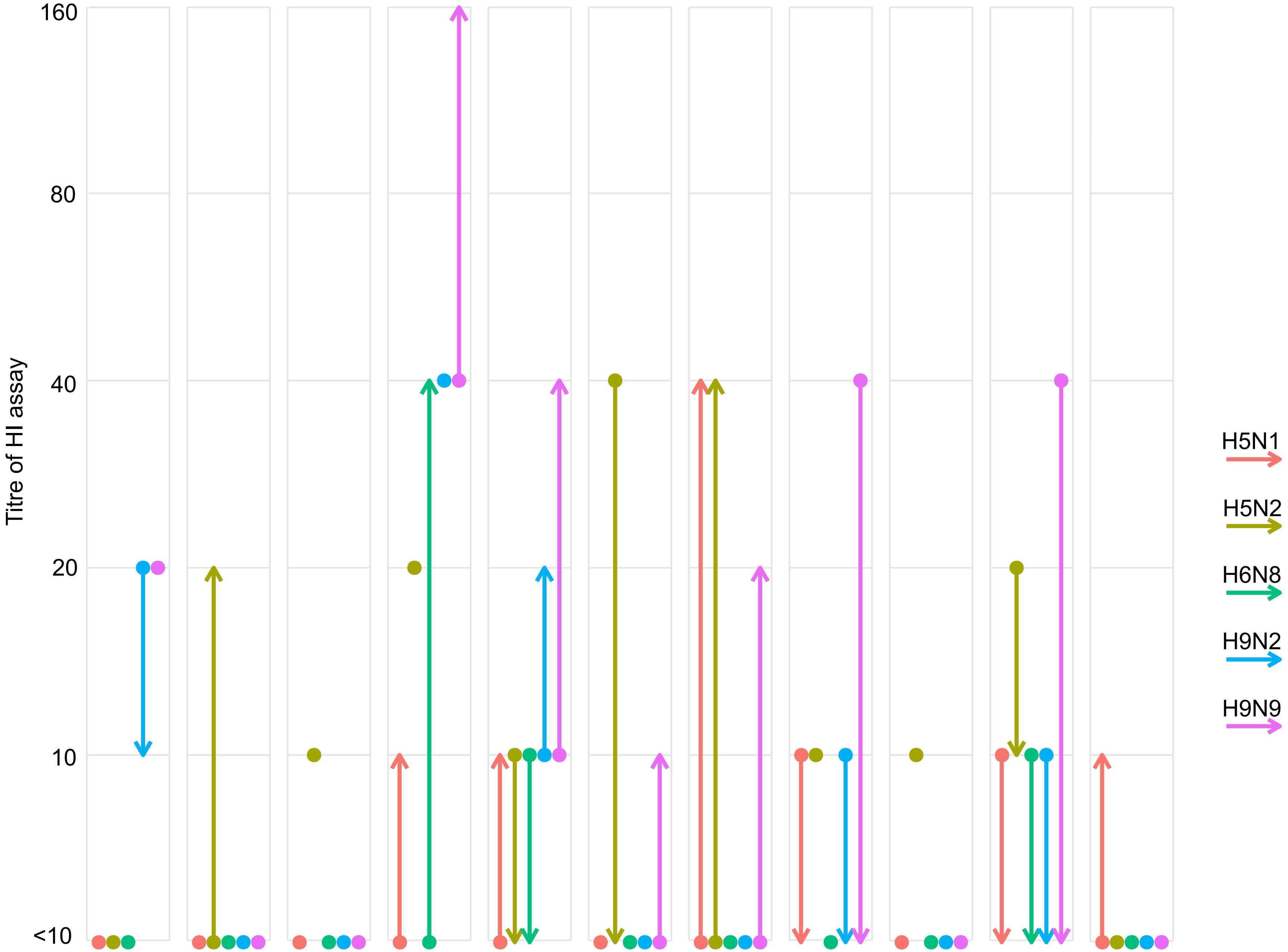

Supplementary Figure 2: Change in HI titre for each antigen (colours) between 2007 and 2008 for each of the eleven birds that were sampled in both years. Dots represent 2007 values, such that lone dots show not change in titre. Arrows show the change from 2007 to 2008. Many titres are not stable between years. Values less than or equal to 10 are considered seronegative in this study.
