# Supplementary material for: Antibody responses to avian influenza viruses in wild birds broaden with age
Source: Proc Biol Sci. 2016 Dec 28;283(1845):20162159. doi: 10.1098/rspb.2016.2159 (PMC5204166; doi:10.1098/rspb.2016.2159)
Supplement: Supplementary Figure 3 [file rspb20162159supp3.pdf]

a)

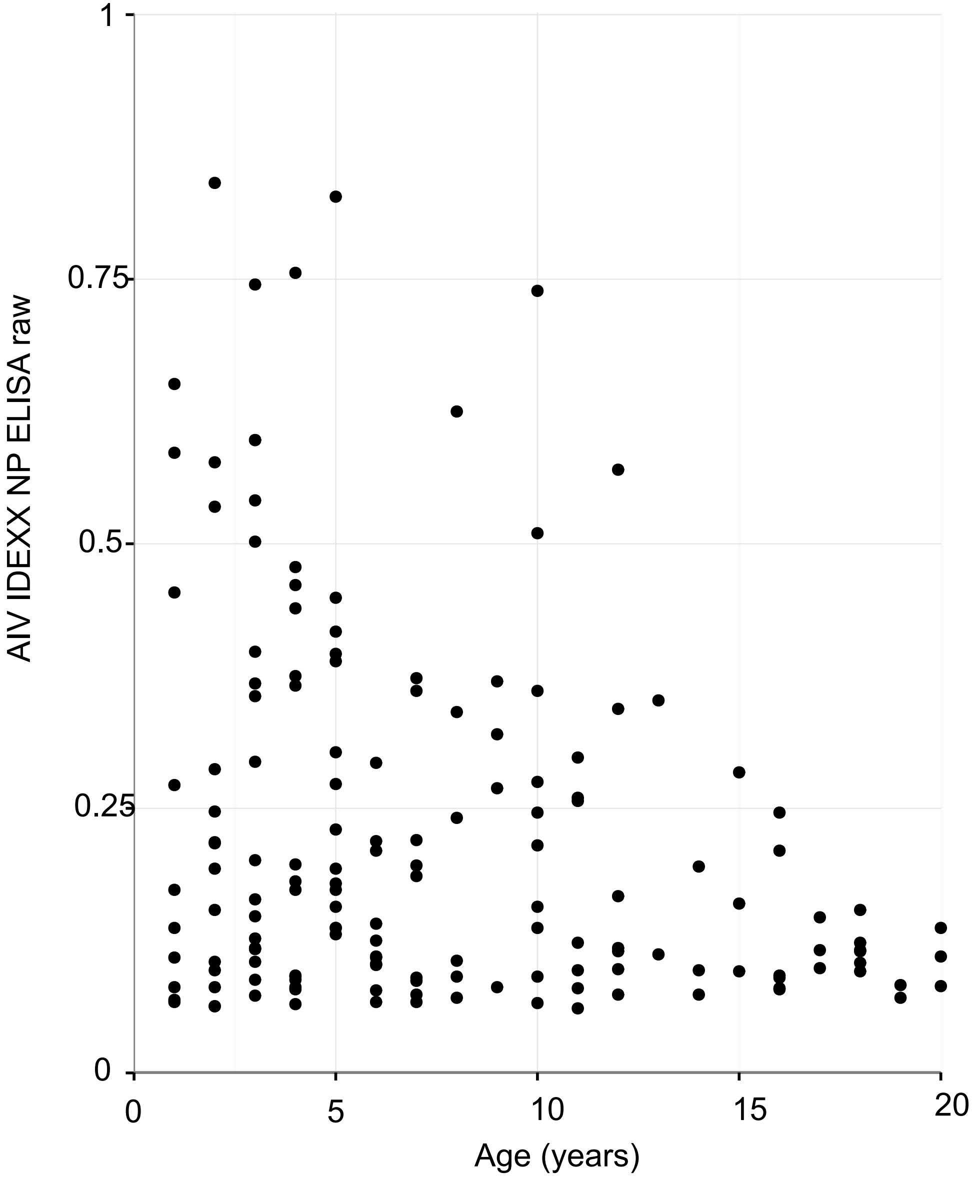

b)

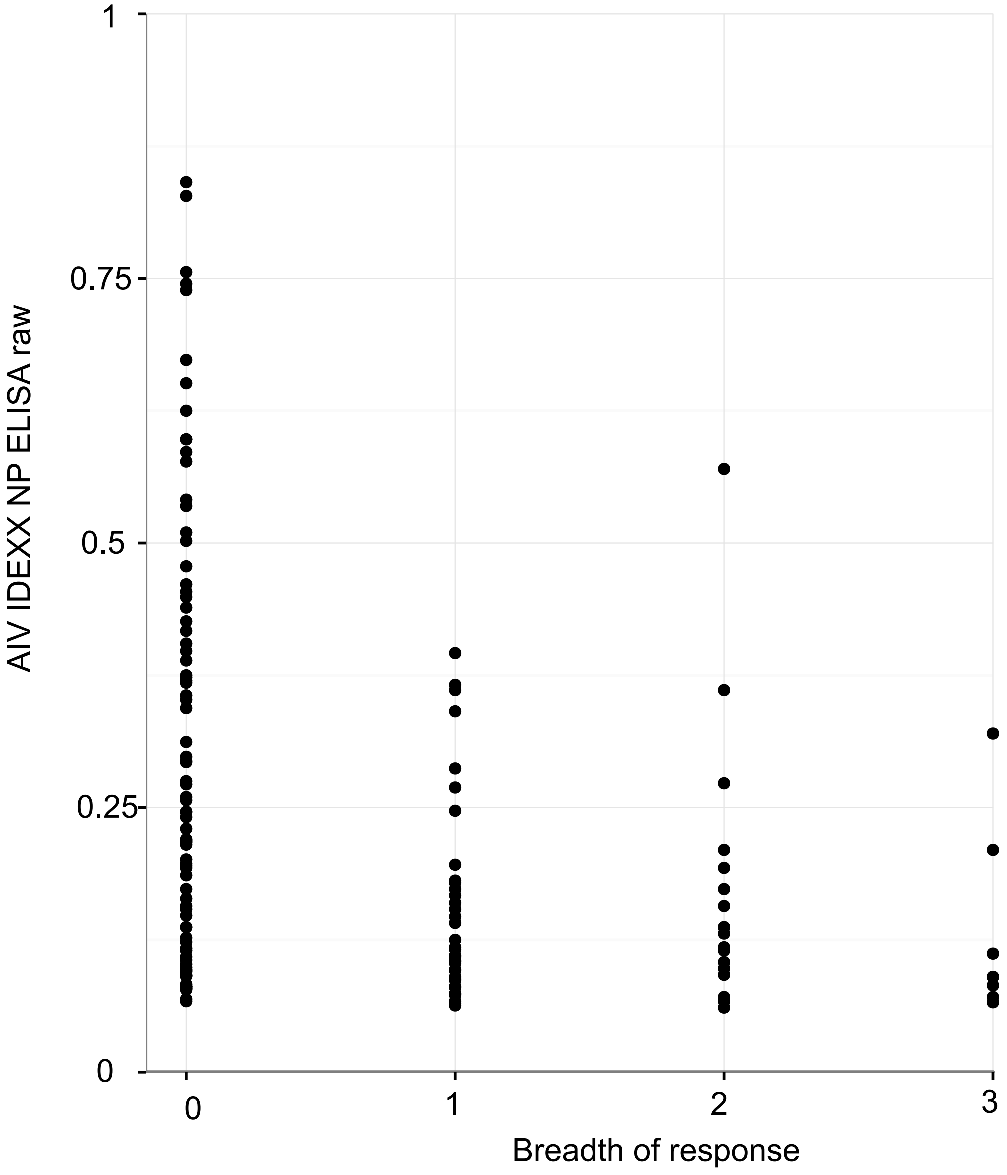

Supplementary Figure 3a: Raw score of NP-ELISA decreases with increasing age.

Supplementary Figure 3b: Raw score of NP-ELISA decreases with increasing breadth of response. Breadth of response calculated as dataset A.
