# Supplementary material for: Antibody responses to avian influenza viruses in wild birds broaden with age
Source: Proc Biol Sci. 2016 Dec 28;283(1845):20162159. doi: 10.1098/rspb.2016.2159 (PMC5204166; doi:10.1098/rspb.2016.2159)
Supplement: Supplementary Tables [file rspb20162159supp4.docx]

**Supplementary Information: Antibody responses to avian influenza viruses in wild birds are age-dependent and broaden with age**

Sarah C Hill ^a^, Ruth J Manvell ^b^, Bodo Schulenburg ^a^, Wendy Shell ^b^, Paul S Wikramaratna ^a^, Chris M Perrins ^a^, Ben C Sheldon ^c^, Ian H Brown ^b^, Oliver G Pybus ^a*^

**Contents:**

**Page 2:** Supplementary Table 1: Ordinal logistic regression models for effect of age on breadth of response.

**Page 3:** Supplementary Table 2: General linear models for individual subtypes.

**Page 4:** Supplementary Table 3: Ordinal logistic regression models for effect of change in AIV IDEXX on change in breadth of response.

**Page 5:** Supplementary Table 4: Generalized linear models for effect of breadth of response on AIV IDEXX.

**Page 6:** Supplementary Table 5: Generalized linear models for effect of age, sex and sample year on AIV IDEXX.

**Supplementary Table 1: Ordinal logistic regression models for effect of age on breadth of response.**

| Dataset | Coefficient estimates (p value) | | |
| --- | --- | --- | --- |
|  | Age | Sample year (2008) | Sex (M) |
| Dataset A (H5N2, H6N8, H9N2) | 0.068 **(p<0.05)** | -0.033 (p=0.927) | -0.885 **(p<0.05)** |
| Dataset B (either H5, H6N8, either H9) | 0.0790 **(p<0.05)** | 0.114 (p=0.750) | -0.601 (p=0.095) |
| Dataset C (responds to both H5N1 and H5N2, H6N8, both H9N2 and H9N9) | 0.062 (p=0.105) | 0.599 (p=0.184) | -0.653 (p=0.158) |
| Dataset D (H5N1, H6N8, H9N9) | 0.081 **(p<0.01)** | 0.579 (p=0.142) | -0.157 (p=0.688) |
| Dataset E (H5N2, H6N8, both H9N2 and H9N9) | 0.068 **(p<0.05)** | 0.077 (p=0.834) | -0.751 **(p<0.05)** |

**Supplementary Table 2: General linear models for individual subtypes.**

| Subtype, best model | Coefficient estimates (p value) | | | |
| --- | --- | --- | --- | --- |
|  | Age | Sample year (2008) | Sex (M) | Interaction effect |
| H5N1, Age+SampleYear+Sex | -0.0449 (p=0.434) | 1.64 **(p=0.0365)** | 0.380 (p=0.494) |  |
| H5N2, Age*SampleYear+Sex | 0.156 **(p=0.0184)** | 1.13 (p=0.111) | -0.768 (p=0.0540) | -0.184 **(p=0.0229)** |
| H6N8, Age+ SampleYear+Sex | 0.111 (p=0.0950) | -0.0539  (p= 0.944) | -1.51 (p=0.167) |  |
| H9N2, Age+ SampleYear+Sex | 0.0959 **(p=0.0190)** | 0.115 (p=0.803) | -1.01 **(p=0.0477)** |  |
| H9N9, Age+ SampleYear+Sex | 0.129 **(p= 0.000154)** | 0.294 (p=0.501) | -0.463 (p=0.303) |  |

**Supplementary Table 3: Ordinal logistic regression models for effect of change in AIV IDEXX on change in breadth of response.**

| Dataset | Coefficient estimates (p value) |
| --- | --- |
|  | Change in AIV IDEXX value between years |
| Dataset A (H5N2, H6N8, H9N2) | -12.657 (0.0859) |
| Dataset B (either H5, H6N8, either H9) | -46.28 **(0.0246)** |
| Dataset C (responds to both H5N1 and H5N2, H6N8, both H9N2 and H9N9) | -10.079 (0.14) |
| Dataset D (H5N1, H6N8, H9N9) | -34.98 **(0.0192)** |
| Dataset E (H5N2, H6N8, both H9N2 and H9N9) | -12.657 (0.0859) |

Note that negative coefficients indicate an association between decreasing raw results of NP ELISA (i.e., *increased* levels of NP antibody) and an increase in breadth of response to different HA types.

**Supplementary Table 4: Generalized linear models for effect of breadth of response on AIV IDEXX**

| Dataset | Coefficient estimates (p value) | |
| --- | --- | --- |
|  | AIV IDEXX ELISA, dataset including all raw ELISA data | AIV IDEXX ELISA, dataset including raw ELISA data <0.5 only* |
| Dataset A (H5N2, H6N8, H9N2) | 1.872 **(6.6e-16)** | 1.353 **(0.000397)** |
| Dataset B (either H5, H6N8, either H9) | 1.954 **(4.62e-07)** | 1.585 **(1.13e-05)** |
| Dataset C (responds to both H5N1 and H5N2, H6N8, both H9N2 and H9N9) | 2.411 **(0.000262)** | 1.930 **(0.000812)** |
| Dataset D (H5N1, H6N8, H9N9) | 2.665 **(8.81e-06)** | 2.385 **(1.05e-05)** |
| Dataset E (H5N2, H6N8, both H9N2 and H9N9) | 1.940 **(1.22e-05)** | 1.418 **(0.000515)** |

*Values <0.5 indicates the bird is seropositive for previous AIV infection by NP-ELISA. This dataset was used to check that the association was not being driven by high ELISA values occurring for birds with no breadth of response, and low ELISA values at breadths greater than 1.

**Supplementary Table 5: Generalized linear models for effect of age, sex and sample year on AIV IDEXX**

| Dataset | Coefficient estimates (p value) | | |
| --- | --- | --- | --- |
|  | Age | Sex (M) | Sample Year (2007) |
| Dataset F (including all raw ELISA data) | 0.182 **(0.00134)** | -1.41 **(0.00366)** | 1.48 **(0.00219)** |
| Dataset G (including raw ELISA data <0.5 only*) | 0.137 **(0.0322)** | -1.01 (0.0794) | -1.160 **(0.0438)** |

*Values <0.5 indicates the bird is seropositive for previous AIV infection by NP-ELISA. This dataset was used to check that the association was not being driven by high ELISA values occurring for birds with no breadth of response, and low ELISA values at breadths greater than 1.
